# Induced Systemic Resistance by a Plant Growth-Promoting Rhizobacterium Impacts Development and Feeding Behavior of Aphids

**DOI:** 10.3390/insects11040234

**Published:** 2020-04-08

**Authors:** Laurent Serteyn, Céleste Quaghebeur, Marc Ongena, Nuri Cabrera, Andrea Barrera, Marco A. Molina-Montenegro, Frédéric Francis, Claudio C. Ramírez

**Affiliations:** 1 Functional and Evolutionary Entomology, Gembloux Agro-Bio Tech, University of Liege, Passage des Déportés 2, B-5030 Gembloux, Belgium; celeste.quagh@hotmail.com (C.Q.); frederic.francis@uliege.be (F.F.); 2 Microbial Processes and Interactions Research Unit, Gembloux Agro-Bio Tech, University of Liege, B-5030 Gembloux, Belgium; marc.ongena@uliege.be; 3 Laboratorio Interacciones Insecto-Planta, Instituto de Ciencias Biológicas, Universidad de Talca, 1141 Talca, Chile; ncabrera@utalca.cl; 4 Laboratorio de Ecología Vegetal, Instituto de Ciencias Biológicas, Universidad de Talca, 1141 Talca, Chile; abarrera@utalca.cl (A.B.); marco.molina@utalca.cl (M.A.M.-M.); 5 Centro de Estudios Avanzados en Zonas Áridas (CEAZA), Universidad Católica del Norte, 1281 Coquimbo, Chile

**Keywords:** *Acyrthosiphon pisum*, *Bacillus amyloliquefaciens*, electropenetrography, *Hamiltonella defensa*, interactions, plant growth-promoting rhizobacteria (PGPR), *Vicia faba*

## Abstract

The effects of microorganisms on plant-insect interactions have usually been underestimated. While plant growth-promoting rhizobacteria (PGPR) are known to induce plant defenses, endosymbiotic bacteria hosted by herbivorous insects are often beneficial to the host. Here, we aimed to assess whether PGPR-induced defenses in broad bean plants impact the pea aphid, depending on its genotype and the presence of endosymbionts. We estimated aphid reproduction, quantified defense- and growth-related phytohormones by GC-MS, and measured different plant growth and physiology parameters, after PGPR treatment. In addition, we recorded the feeding behavior of aphids by electropenetrography. We found that the PGPR treatment of broad bean plants reduced the reproduction of one of the pea aphid clones. We highlighted a phenomenon of PGPR-induced plant defense priming, but no noticeable plant growth promotion. The main changes in aphid probing behavior were related to salivation events into phloem sieve elements. We suggest that the endosymbiont *Hamiltonella defensa* played a key role in plant-insect interactions, possibly helping aphids to counteract plant-induced resistance and allowing them to develop normally on PGPR-treated plants. Our results imply that plant- and aphid-associated microorganisms add greater complexity to the outcomes of aphid-plant interactions.

## 1. Introduction

First defined by Hiltner in 1904, the rhizosphere is “the soil compartment influenced by the root”, rich in microbial activity [1]. The so-called rhizobacteria interact with each other and with the plant [2]. Rhizobacteria that exert beneficial effects on plant development are called plant growth-promoting rhizobacteria (PGPR) [3]. Some non-pathogenic rhizobacteria can induce in the plant a systemic resistance (ISR) to fungal, bacterial, or viral diseases, but also to insect and nematode pests [4,5,6].

Due to phylogenic confusion, an “operational group *Bacillus amyloliquefaciens*” was proposed to include the soil-borne *B. amyloliquefaciens* and the plant-associated *Bacillus siamensis* and *Bacillus velezensis* [7]. This group of PGPR, hereafter termed “*B. amyloliquefaciens”*, has a direct effect on plant growth, improving the nutritional status of plants and modulating the production of phytohormones [8]. Previous studies have shown that *B. amyloliquefaciens* triggers induced systemic resistance (ISR) in several plant species [9], which reduces the fitness of herbivore insects [10,11,12]. Lipopeptides (fengycin and surfactin) are elicitors produced by the PGPR genus *Bacillus*, and are involved in the induction of plant resistance [6]. After the perception of these elicitors by root cells, pathways regulated by jasmonic acid (JA) and ethylene are activated, resulting in the chemical priming of the plant [5,6,13,14]. Therefore, the capacity of primed plants to mobilize defense responses is lastingly augmented, and defense responses only occur once the pathogen or the pest attacks [15,16]. Previous studies have reported negative impacts of PGPR on insect development (Lepidoteran pests and aphids), sometimes accompanied by a promotion of plant growth, which balances the effect of pest invasion [17,18].

The pea aphid, *Acyrthosiphon pisum* Harris is an important pest of Fabaceae crops (peas, alfalfa, beans, etc.). This insect causes direct damage by consuming plants, and indirect impacts by transmitting phytoviruses [19]. The alfalfa race, whose main host is *Medicago sativa* L., is one of the three most predominant biotypes of *A. pisum* in Chile [20]. The bacterial endosymbiont community of *A. pisum* is well described [21]. Facultative endosymbionts can provide adaptive advantages for their host, in terms of digestion, fecundity, and resistance to abiotic or biotic stresses [22], including stresses due to induced plant defenses [23]. Among these endosymbionts, *Hamiltonella defensa* confers protection against parasitoids and attenuates the emission of plant defensive volatiles following aphid attack [24,25]. Also, growing evidence supports the idea that *H. defensa* manipulates the salivary compounds and feeding behavior of its host, influencing plant-insect interactions [23,26,27].

Regarding the literature, the combined quadripartite interactions between plants, herbivorous insects, their endosymbionts, and plant-associated microorganisms, remain largely unexplored. Enlightenment regarding such interactions could guide the development of environment-friendly tools to protect plants against pests, and thus increase crop yields. In this study, we aimed to assess whether PGPR-induced defenses in broad bean plants impact the pea aphid, depending on its genotype and the presence of endosymbionts. Therefore, the population and feeding behavior of two distinct pea aphid genotypes of the alfalfa biotype—also differentiated by the presence of *H. defensa*—in response to plant defense priming caused by the inoculation of *B. amyloliquefaciens* FZB42 on *Vicia faba* L. were studied.

## 2. Materials and Methods

### 2.1. Plants, Insects and Rhizobacteria

Organic broad beans (cv. “Anka Mapu”) (Anka Mapu Organic Farm, San Clemente, Chile) were employed for all of the experiments and aphid rearing. To ensure that no other soil bacteria could interact with the broad bean roots, all seeds were cleaned and sterilized.

Two different clones of the alfalfa biotype of *A. pisum* Harris were collected in alfalfa fields (*Medicago sativa* L.) in Central Chile (Linares and Panguilemo, both in Maule Region, hereafter named “+E” and “−E”, respectively) and maintained on broad beans in a culture room (21 ± 1 °C, 60% RH, 16 h light/8 h dark) in the Laboratorio de Interacciones Insecto-Planta (Universidad de Talca, Chile).

The FZB42 strain of *B. amyloliquefaciens*, considered as a heterotypic synonym of *B. velezensis* [28], was kindly provided by Prof. R. Borriss of Humboldt University, Berlin, and was cryopreserved at −80 °C in glycerol 20% (v:v). Bacteria were grown in lysogeny broth (Merck, Darmstadt, Germany) in a shaker-incubator at 30 °C with 200 rpm agitation for 24 h. The optical density of the bacterial solutions was measured with a spectrophotometer (Biochrom WPA, model: Biowave DNA; Cambridge, UK) at 600 nm.

### 2.2. Aphid Genotype and Endosymbiont Screening

Aphid DNA extraction and PCR were performed following the protocols of Peccoud et al. (2008) [20], with minor modifications. Aphids were genotyped using nine microsatellite loci (*ApF08*, *ALB04*, *ALB08*, *ALB12*, *ALB07*, *ApH10*, *Ap03*, *ApH08*, and *ALA12*), using the M13 labeling technique with fluorescent dyes described by Schuelke (2000), followed by automated fragment analysis by Macrogen Inc. (Seoul, Korea) [29]. Allele sizes were determined using GENEMARK version 1.3 [30].

In order to check the occurrence of bacterial endosymbionts in each clone, the procedure described by Peccoud et al. (2014) [31] was followed, using two Multiplex PCRs on the whole-body DNA extracts of the aphids. The investigated endosymbionts were *Buchnera aphidicola*, *Spiroplasma* sp., *Regiella insecticola*, *Hamiltonella defensa*, *Rickettsiella* sp., pea aphid X-type symbiont (PAXS), *Serratia symbiotica*, and *Rickettsia* sp.

### 2.3. Aphid Population Growth According to PGPR Inoculation

Twelve-day-old broad beans (with two fully developed leaves, stage 12 on the BBCH-scale) were treated with 15 mL of bacterial solution at an average OD600 of 5 × 10^8^ cells mL^−1^. This bacterial solution (“+PGPR”)—or the same volume of water, as a control treatment (“−PGPR”)—was applied with a syringe onto the surface of the soil. Seven days after soil inoculation, five 10-day-old aphid nymphs were placed (Day 0) and confined on individual plants (n = 9 for each of the 6 treatments: “no aphid”, “+E”, and “−E”, with or without PGPR). The systems were placed in a culture room (20 ± 2 °C, 60 ± 5 % RH, 16 h light/8 h dark). All aphids were counted after 6 days, and then removed from the plants to perform measures of plant parameters. In addition, plant physiology performance was estimated by measuring the photochemical efficiency of photosystem II (Fv/Fm) and the electron transport rate in the active reaction centers (ET_0_/RC), using a chlorophyll fluorimeter (Hansatech Pocket PEA; Pentney, UK) on *V. faba* leaves (n = 9).

Samples of leaves were collected 6 days after aphid infestation (Day 6) to perform hormone profile analysis. For each of the 6 treatments, 3 plants of the 9 replicates were randomly selected, and two contiguous medium leaves were harvested and stored at −80 °C after instant freezing in liquid nitrogen. Lyophilisation, phytohormone extraction, derivatization, and GC-MS analyses were carried out following the procedure of Ramos et al. (2018) [32] and Carrasco Loba et al. (2017) [33]. The concentration of three phytohormones in leaf extracts was obtained, expressed in picomoles per gram of fresh weight: salicylic acid (SA), jasmonic acid (JA), and indole-3-acetic acid (IAA).

Different plant traits were also evaluated at Day 0 and Day 6. The specific leaf area (SLA) was measured on broad bean plants not used for hormone profile analysis (n = 6; 2 leaves per plant) [34]. The shoots and roots of these plants were separated, dried, and then weighted to evaluate the dry weights of the roots and the total biomass.

### 2.4. Feeding Behavior of Aphids

To study the feeding behavior of aphids on PGPR-inoculated plants, an electrical penetration graph (EPG) was conducted on the two clones +E and −E at Day 0. A 25 μm-thin and 2 cm-long gold-wire was attached to the adult aphid’s dorsum with a small droplet of conductive water-based silver glue (colloidal silver, Ted Pella Inc., Redding, USA, CA). Then, the aphids were left for 30 min on moistened filter paper in a Petri dish for conditioning. Afterwards, the aphids were placed on broad bean leaf in a Faraday cage and connected with the probe of a DC−EPG (EPG Systems, Wageningen, The Netherlands) by a copper nail. EPG signals were recorded over 4 h with the Stylet^+^d software (EPG Systems). A 4-channel amplifier (model Giga-4) was used to carry out recordings for each treatment. Each aphid was recorded only once, and 27, 31, 27, and 26 replicates were performed for the −E/−PGPR, −E/+PGPR, +E/−PGPR, and +E/+PGPR treatments, respectively. The obtained waveforms were identified with the Stylet^+^a (EPG Systems) and A2EPG softwares [35]. The sequences of the waveforms for each replicate were imported into the Excel workbook of Sarria et al. (2009) [36] to automatically calculate all of the sequential and non-sequential EPG variables that characterize the probing and ingestion phases, among which 65 were selected for further statistical analyses.

### 2.5. Statistical Analyses

The total number of nymphs, 6 days after aphid infestation, was analyzed by generalized linear models (GLMs) using R software, with two factors: PGPR treatments (+PGPR and −PGPR) and aphid clones (+E and −E). Because GLMs with a Poisson distribution of errors were over-dispersed, a quasi-Poisson distribution was used. As the winged form of adults appeared differentially between clones, being particularly high in the +E clone, the number of final alate adults was included in the GLM as a co-variable.

In order to find relevant EPG parameters varying among treatments, the 65 EPG parameters were subjected to forward stepwise discriminant analysis in the STATISTICA software. Those parameters significantly discriminating between treatments were subjected to a MANOVA to identify differences in mean values.

Differences in the mean contents of JA, SA, and IAA and in plant growth parameters (Fv/Fm, ET_0_/RC, SLA, root dry weight, and total biomass) among treatments were tested with a GLM with a Gaussian error distribution, also using R software.

In all cases, multiple pairwise comparisons were made using Tukey’s honestly significant difference (HSD) test under the “multcomp” package.

## 3. Results

**Figure A1:**
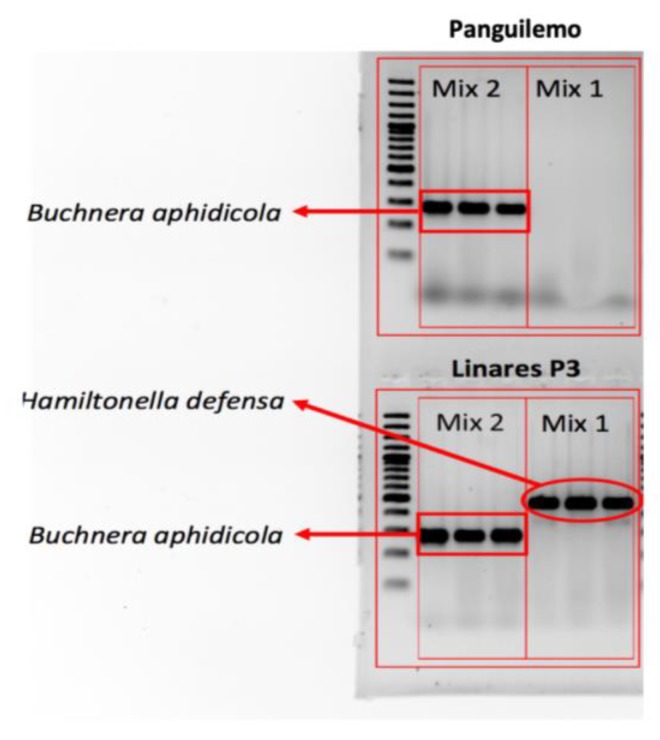
The PCR screening of endosymbionts in *A. pisum* clones −E (above) and +E (below).

The Panguilemo clone (hereafter named “−E”) only harbored the primary endosymbiont *Buchnera aphidicola*, while the Linares clone (“+E”) harbored *B. aphidicola* along with the facultative endosymbiont *Hamiltonella defensa* (see Supporting Information, Appendix A, Figure A1). The allele sizes of the nine microsatellite loci, for each clone, are presented in Table 1.

**Figure 1:**
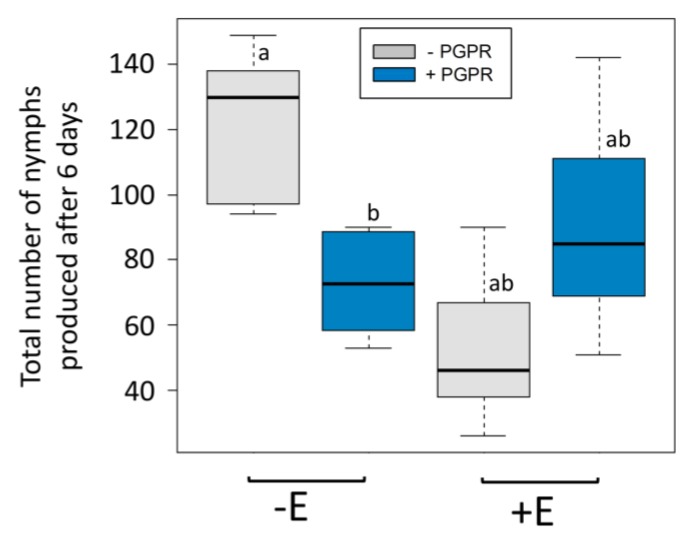
Boxplots of the numbers of nymphs produced 6 days after infestation with five *A. pisum* aphids −E and +E (absence and presence of endosymbiont *Hamiltonella defensa*, respectively), on plants inoculated or not inoculated with *Bacillus amyloliquefaciens* (+plant growth-promoting rhizobacteria (PGPR) and −PGPR, respectively). The different letters indicate significant differences between groups (Tukey’s honestly significant difference (HSD) *p* < 0.05, n = 9).

The PGPR treatment of plants affected the reproduction of aphids, depending on the clone (interaction effect: D = 51.53; df = 1, 30; *p* < 0.001), with facultative symbiont-free aphids (“−E”) exhibiting significantly lower reproduction on PGPR-treated plants (“+PGPR”) than on control plants (“−PGPR”), whereas the reproduction of facultative symbiont-hosting aphids (“+E”) was not significantly affected by PGPR treatments (Figure 1). Globally, aphid reproduction on broad bean was lower for +E aphids (main effect: D = 71.58; df = 1, 33; *p* < 0.001), and PGPR treatment did not significantly affect aphid reproduction (main effect: D = 3.34; df = 1, 32; *p* = 0.477).

**Figure 2:**
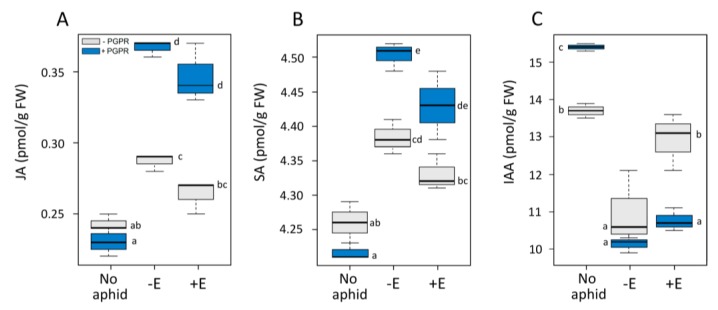
Boxplots of phytohormone levels (jasmonic acid (JA), salicylic acid (SA), and indole-acetic acid (IAA)) in plants inoculated or not inoculated with *B. amyloliquefaciens* (+PGPR and −PGPR, respectively) and infested or not infested by *A. pisum* aphids −E and +E (absence and presence of endosymbiont *H. defensa*, respectively). The different letters indicate significant differences between groups (Tukey’s HSD, *p* < 0.05, n = 3).

Jasmonic acid (JA) and salicylic acid (SA) contents were not affected by PGPR treatment in non-aphid-attacked plants (Figure 2A,B). Both phytohormones were induced in plants attacked by either −E or +E aphids, and contents were even higher in plants treated with both PGPR and aphids, with no significant differences between −E and +E aphids (Figure 2A,B). Conversely, the indole-acetic acid (IAA) content was significantly higher in +PGPR than in −PGPR plants for non-aphid-attacked plants, lower in +PGPR than in −PGPR plants attacked by +E aphids, and similarly low in +PGPR and −PGPR plants attacked by −E aphids (Figure 2C).

**Figure 3:**
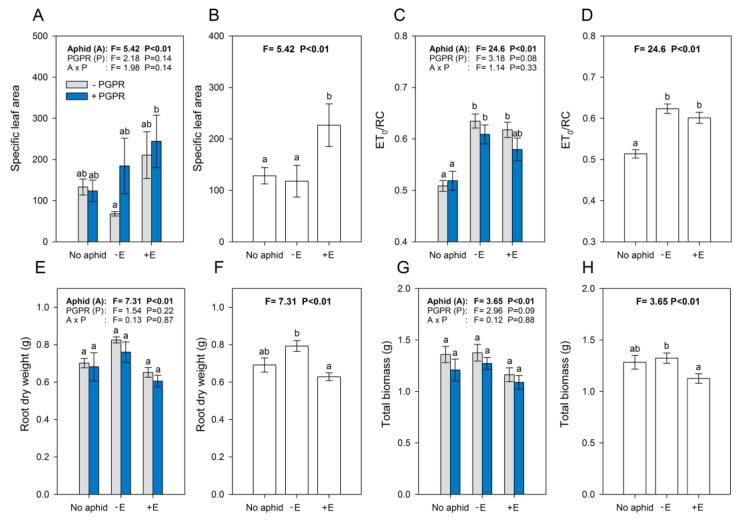
The responses of plants inoculated or not inoculated with *B. amyloliquefaciens* (+PGPR and −PGPR, respectively) and infested or not infested by −E and +E *A. pisum* aphids (absence and presence of the endosymbiont *H. defensa*, respectively). Panels (**A**,**C**,**E**,**G**); and (**B**,**D**,**F**,**H**) show all effects (aphid, PGPR, and interaction) and aphid effects (as the only significant in all cases), respectively. The different letters indicate significant differences between groups (Tukey’s HSD, *p* < 0.05). Error bars represent the standard error of mean (n = 9 for the ET_0_/RC; n = 6 for others).

PGPR treatment did not significantly affect the specific leaf area (SLA; F = 2.18; df = 1; *p* = 0.14; Figure 3A), electron transport rate in the active reaction centers (ET_0_/RC; F = 3.18; df = 1; *p* = 0.08; Figure 3C), root dry weight (F = 1.54; df = 1; *p* = 0.22; Figure 3E), or total biomass (F = 2.96; df = 1; *p* = 0.09; Figure 3G). Independently of the PGPR treatment, plants attacked by +E aphids exhibited a higher SLA than plants attacked by −E aphids and non-attacked plants (F = 5.42; df = 2; *p* < 0.01; Figure 3B). Contrastingly, the ET_0_/RC was significantly higher in plants attacked by both +E and −E aphids compared to in the controls without aphids (F = 24.6; df = 2; *p* < 0.01; Figure 3D). Root dry weight (F = 7.31; df = 2; *p* < 0.01; Figure 3F) and total biomass (F = 3.65; df = 2; *p* < 0.01; Figure 3H) were both affected by aphid treatments, with plants attacked by +E aphids showing lower values than −E, independently of PGPR treatment. The photochemical efficiency of photosystem II (Fv/Fm) was not affected by endosymbionts, PGPR, or by their interaction (data not shown).

The discriminant electropenetrography (EPG) parameters (Table 2) revealed significant differences in the global probing behavior among treatments (Wilks’ lambda = 0.39, *p* < 0.01). Both aphid clones, probing on +PGPR plants in comparison with non-primed plants (−PGPR), showed a longer time from the beginning of that probe to the first sieve element activities and to the first sustained phloem ingestion (E2), and a shorter duration and proportion of time spent on xylem activities (G). Besides these similarities, interesting opposite behaviors between aphids occurred: +E aphids on +PGPR plants presented a shorter duration of first probe, higher number of salivations into the sieve element events (E1), longer mean duration of E1, shorter duration of non-probing (NP) just after the probe of the first sustained E2, longer time from the beginning of the first probe to the first potential drop (pd), longer mean duration of NP, and longer duration of the E1 event followed by the first E2, than those on −PGPR plants.

## 4. Discussion

In this study, we highlighted the phenomenon of PGPR-induced plant defense priming. This induced systemic resistance (ISR) reduced the reproduction of only one of the two pea aphid clones. Additionally, it seemed that the feeding behavior of this clone played a crucial role in counteracting plant defenses.

In order to explain the observed influence of PGPR on aphid clone development, we aimed to understand how PGPR treatment affected the plant’s physiology. *Bacillus amyloliquefaciens* induced an ISR in broad beans, because the highest contents of JA were measured in aphid-colonized plants [9]. Interestingly, SA contents were similarly enhanced by PGPR treatment, as mentioned in previous studies [37,38]. Phytohormone contents were not increased by PGPR treatment in aphid-free plants, a cost−Effective phenomenon called “plant defenses priming” that makes plants more reactive when attacked by a pest or a pathogen. Other evidence of a PGPR-induced systemic resistance caused by a JA-dependent signaling pathway has been highlighted [39,40,41]. It is noteworthy that, in our study, JA and SA levels did not show signs of negative crosstalk, as reported in other aphid-plant systems [42].

Several studies have reported various effects of PGPR on herbivorous insect development, including neutral [43,44], negative [17,45,46,47], and positive effects [48,49]. Hence, generalizing the effects of PGPR on aphid populations seems to be risky because responses are highly dependent on the set of involved species [47]. To our knowledge, the only study on *B. amyloliquefaciens* FZB42 and aphids had shown an alteration of the life traits of the aphid *Brevicoryne brassicae* on PGPR-treated calabrese plants [46], which is consistent with our observation of the decreased reproduction of *A. pisum* −E on PGPR-treated broad beans.

One of our most striking results was the differential development of the two pea aphid clones on PGPR-treated plants. Such a difference could be due to genetic factors, on the one hand, or to the symbiont profile, on the other hand. According to the study of Peccoud et al. (2008), aphids that were sampled on the same host plant in the field should show more similar microsatellite profiles [20]. Another study highlighted a variable response of plant defenses, and their effects on aphid development, according to the biotype of the pea aphid: on alfalfa plants, the alfalfa biotype induced the defenses to a lesser extent and performed better than the pea biotype [50]. In our case, both clones belonged to the alfalfa biotype and were reared on broad beans. Additionally, independently of PGPR treatment, their population growth and induction of plant defenses were similar between the two clones. Therefore, we believe that the presence of facultative endosymbionts had a greater influence on plant-insect−PGPR interactions than the genotype of the aphids. To validate this hypothesis, more experiments should be carried out on the two same genotypes, but with comparable symbiont profiles.

Our results suggest that such symbionts add greater complexity to the outcomes of plant-centered multitrophic interactions. Among the bacterial endosymbionts of *A. pisum*, *H. defensa* is known to provide resistance for aphids against the parasitoid *Aphidius ervi*, with some variability according to symbiont strain [51]. Besides, *H. defensa* allows whiteflies to suppress JA-regulated defenses thanks to effectors in the insect saliva, which might be symbiont-borne [23]. Moreover, *H. defensa* was able to attenuate the emission of plant defensive volatiles following aphid attack [25]. In our study, SA and JA contents were slightly lower in plants colonized by +E aphids than in those colonized by −E aphids, and were not significantly different between non-primed plants attacked by +E aphids and non-primed aphid-free plants. These two observations, taken together, support the hypothesis that, instead of directly strengthening the aphid, *H. defensa* helps aphids to inhibit plant defenses [23,25], eventually allowing the pest to develop normally on primed plants. Literature suggests that *H. defensa* indirectly counteracts the plant ISR, by manipulating aphid physiology and behavior [25]. To validate this hypothesis, the same aphid genotype should be exposed to antibiotics, and then micro-injected with *H. defensa* [52], to enlighten regarding the effect of the sole presence of the endosymbiont. Our work on field aphid populations was closer to real conditions, though, without artificial manipulations that could also introduce biases.

Along with plant defense induction, *B. amyloliquefaciens* is known to promote plant growth [8], which could influence aphid development parameters. Mainly implicated in plant growth, the auxin IAA also provides adaptive responses to abiotic and biotic stresses, including insect feeding [53]. Some PGPR can produce IAAs, resulting in higher levels of this hormone in plant tissues [54,55,56], as observed in our study for aphid-free plants. Globally, we found that IAA contents were reduced when aphids colonized the plants, a phenomenon also observed with *Spodoptera exigua* on peanut and tomato plants [57]. However, when they added PGPR on plants, even higher levels of IAA were usually observed, unlike in our study—especially concerning +E aphids—where lower contents of IAA were observed in PGPR-treated plants, compared to in the non-primed plants.

Even if the IAA contents varied between plant treatments, plant inoculation with PGPR did not promote great changes in plant growth features. This is surprising because, as is the case with many plants [58], PGPR is usually known to promote the growth of broad bean plants [59,60]. Therefore, differences in the development of −E and +E aphids facing PGPR-treated plants could not be attributed to differences in plant physiological states. Independently of PGPR treatment, plants colonized by +E aphids showed a larger SLA, and a reduced root dry weight and total biomass, compared to −E aphid-colonized plants. Hackett et al. (2013) stated that the allocation of biomass to roots was reduced in potatoes colonized by the aphid *Macrosiphum euphorbiae* harboring *H. defensa*, compared to in plants attacked by *H. defensa*-free aphids or aphid-free plants [27]. Both our results and theirs support the idea that *H. defensa* imposes higher nutritional demands on its host, resulting in a higher phloem uptake by aphids, which would provoke a higher compensatory photosynthetic activity, eventually resulting in resource allocation to the stem and leaves, instead of roots. Further research is needed to clarify the effects of such quadripartite interactions on more growth phytohormones and on growth parameters, based on longer experimental duration.

It had been suggested above that presence of *H. defensa* in aphids may result in modified feeding behavior. Indeed, the earliest steps of feeding behavior of the aphid *Aphis craccivora* have been reported to change in the presence of *H. defensa* [26]. To help in identifying both the defense mechanisms with the most impact on aphid development, and the strategies implemented by aphids to counteract them, EPG parameters can be subdivided into various locations of plant defense factors: the plant surface, mesophyll cells (intercellular and intracellular), and phloem sieve elements [61]. Interestingly, only a few parameters associated with surface factors were significantly modified by the PGPR treatment, suggesting that no structural changes of the plant epidermis, nor of repulsive volatile organic compounds, occurred at this stage of plant priming [62]. Both aphid clones showed disturbance in the mesophyll phases on primed plants, such as longer times from the beginning of the probe to first E1 and E2 [63]. Beside these common behavioral traits between the two clones, opposite reactions to plant priming were observed. First, +E aphids on +PGPR performed shorter first probes and had longer times from the first probe to the first epidermis/mesophyll cell penetration, than those on −PGPR plants. This suggests that +E aphids were perturbed by plant priming at very early phases of feeding. Second, +E aphids increased the number of E1 events in primed plants. Even if the EPG experiment took place at a very early stage of aphid infestation (no detectable change in phytohormone contents), we suppose that these aphids were rapidly confronted with the occlusion of sieve elements, and then interrupted phloem ingestion, and started secreting watery saliva [64]. Third, +E aphids salivated longer in the phloem before the first E2 on +PGPR plants. Because various jasmonate components can be detected in the phloem and influence aphid feeding behavior [65], it seems that +E aphids were able to recognize the primed state of *V. faba* once it reached the phloem sap, and to adapt, in consequence, its salivation behavior. In *A. pisum*, some salivary proteins were identified as effectors that modulate insect feeding and survival on *V. faba* [66,67], and potentially inhibit plant immune responses in phloem sieve elements [64,68,69]. A reduction of salivation duration is typically a negative reaction to phloem-based antixenosis resistance [63]. Therefore, those longer salivation events before food ingestion have probably attenuated the ISR, allowing +E aphids to exhibit normal reproductive performances. Such higher salivation in the phloem could also enhance the activity of symbiont-borne salivary effectors [23]. Unfortunately, we could not corroborate the hypothesis that +E aphids ingested more phloem [27], because of the too-short duration of EPG recordings. Indeed, most of E2 events were interrupted by the ending of the recordings, making all of the EPG parameters related to phloem ingestion hardly exploitable. More studies are needed to identify the effects of different genotypes of the aphid, within the same biotype, on its feeding behavior when exposed to plant defenses, and to clarify the role played by *H. defensa* in these changes of feeding behavior.

## 5. Conclusions

The roles of PGPR in insect-plant interactions are still unclear and deserve more attention from entomologists, phytologists, and microbiologists. Additionally, as seen here, insect-related microorganisms, such as the endosymbiont *H. defensa*, add greater complexity to the process of drawing strong conclusions. Therefore, such quadripartite interactions deserve further study, to assess, for instance, how various taxa of symbiotic bacteria and PGPR determine the fates of various species and genotypes of interacting plants and insects.

## Figures and Tables

**Table 1:** The multilocus genotypes of the −E and +E clones of *Acyrthosiphon pisum*. The numbers represent the allele sizes (in bp).

Clone					*Locus*				
	ApF08	ALB04	ALB08	ALB12	ALB07	ApH10	Ap03	ApH08	ALA12
+E	183/189	267/269	277/290	321/345	136/152	203/213	261/261	268/284	435/459
−E	181/193	266/268	294/302	329/335	135/171	215/217	254/261	266/268	449/457

**Table 2:** The mean ± SD of significantly discriminant electrical penetration graph (EPG) parameters, for −E and +E clones of *A. pisum* on *B. amyloliquefaciens*-treated or control plants. NP: non-probing; C: stylet pathway phase; pd: potential drop related to cell punctures; G: xylem ingestion; F: difficulties during stylet penetration; E1: salivation into sieve elements; E2: phloem ingestion; E: phloem phase including E1 and E2. The different letters within rows indicate statistically differences (*p* < 0.05).

EPG Parameters	Localization of Resistance Factors			−E		+E	
	Surface	Mesophyll	Phloem	−PGPR	+PGPR	−PGPR	+PGPR
Mean duration of NP (min)	X			6.41 ± 5.95 ^d^	5.59 ± 9.00 ^c^	2.97 ± 1.76 ^a^	4.27 ± 5.21 ^b^
Duration of the 2nd non-probe period (min)	X	X		4.31 ± 5.50 ^d^	2.99 ± 3.74 ^c^	2.69 ± 3.80 ^b^	1.88 ± 2.01 ^a^
Number of short probes (C < 3 min)	X	X		5.59 ± 5.02 ^a^	5.97 ± 5.33 ^a^	10.15 ± 10.84 ^b^	8.15 ± 9.59 ^b^
Time from start of EPG to 1st E2 (min)	X	X	X	129.37 ± 77.09 ^d^	123.35 ± 76.93 ^b^	127.98 ± 86.23 ^c^	103.35 ± 68.26 ^a^
Duration of NP just after the probe of the 1st sustained E2 (min)	X		X	1.39 ± 3.90 ^c^	1.82 ± 4.82 ^d^	0.73 ± 2.12 ^b^	0.32 ± 0.83 ^a^
Mean duration of F (min)		X		0.10 ± 0.49 ^a^	0.65 ± 3.28 ^b^	4.40 ± 16.31 ^c^	0.00 ± 0.00ab ^c^
Time from the beginning of the 1st probe to 1st pd (min)		X		6.98 ± 18.80 ^d^	4.80 ± 12.57 ^c^	0.79 ± 1.75 ^a^	1.06 ± 2.57 ^b^
Duration of 1st probe (min)		X	X	10.90 ± 44.98 ^b^	33.57 ± 76.30 ^d^	18.43 ± 63.67 ^c^	1.17 ± 1.95 ^a^
Time from the beginning of that probe to 1st E (min)		X		22.09 ± 14.57 ^b^	31.02 ± 31.60 ^d^	21.36 ± 17.44 ^a^	25.13 ± 12.45 ^c^
Duration of 1st E (min)			X	71.97 ± 73.17 ^d^	56.78 ± 61.83 ^a^	70.14 ± 80.36 ^c^	60.10 ± 68.13 ^b^
Time from the beginning of that probe to 1st sustained E2		X	X	25.05 ± 17.09 ^b^	31.39 ± 32.46 ^d^	22.40 ± 19.16 ^a^	25.79 ± 13.09 ^c^
Mean duration of E1 (min)			X	1.14 ± 0.86 ^a^	1.10 ± 1.58 ^a^	1.05 ± 1.14 ^a^	1.38 ± 1.49 ^b^
Number of E1			X	1.22 ± 0.97 ^a^	1.42 ± 1.06a ^b^	1.33 ± 1.18 ^a^	2.31 ± 2.04 ^b^
Duration of E1 followed by the 1st E2 (min)			X	1.04 ± 0.72 ^c^	0.92 ± 0.64 ^b^	0.75 ± 0.51 ^a^	0.93 ± 0.52 ^b^
Duration of E1 followed by the 1st sustained E2 (min)			X	1.12 ± 0.90 ^b^	0.86 ± 0.53 ^a^	0.93 ± 1.09 ^a^	1.14 ± 1.10 ^b^
Number of sustained E2 (<10 min)			X	1.00 ± 0.78 ^a^	1.03 ± 0.75 ^a^	0.89 ± 0.75 ^a^	1.65 ± 1.35 ^a^
Duration of G (min)				13.65 ± 30.82 ^c^	5.98 ± 15.32 ^a^	18.85 ± 46.92 ^d^	6.94 ± 19.64 ^b^
Number of G				0.30 ± 0.67 ^a^	0.29 ± 0.78 ^a^	0.37 ± 0.69 ^a^	0.12 ± 0.33 ^a^
% of probing spent in G				7.44 ± 16.92 ^b^	3.35 ± 8.32 ^a^	8.70 ± 21.19 ^b^	3.31 ± 9.45 ^a^

## References

[1] Hartmann A., Rothballer M., Schmid M. (2008). Lorenz Hiltner, a pioneer in rhizosphere microbial ecology and soil bacteriology research. Plant Soil.

[2] Dardanelli M.S., Medeot D.B., Paulucci N.S., Bueno M.A., Vicario J.C., García M., Bensi N.H., Niebylski A.M., Malik A., Grohmann E. (2012). Biochemical Processes of Rhizobacteria and Their Application in Biotechnology.

[3] Kloepper J.W., Rodríguez-Kábana R., Zehnder G.W., Murphy J.F., Sikora E., Fernández C. (1999). Plant root-bacterial interactions in biological control of soilborne diseases and potential extension to systemic and foliar diseases. Australas. Plant Pathol..

[4] Ramamoorthy V., Viswanathan R., Raguchander T., Prakasam V., Samiyappan R. (2001). Induction of systemic resistance by plant growth promoting rhizobacteria in crop plants against pests and diseases. Crop Prot..

[5] Walters D., Heil M. (2007). Costs and trade-offs associated with induced resistance. Physiol. Mol. Plant Pathol..

[6] Ongena M., Jacques P. (2008). *Bacillus* lipopeptides: Versatile weapons for plant disease biocontrol. Trends Microbiol..

[7] Fan B., Blom J., Klenk H.P., Borriss R. (2017). *Bacillus amyloliquefaciens*, *Bacillus velezensis*, and *Bacillus siamensis* form an “Operational group *B. amyloliquefaciens*” within the *B. subtilis* species complex. Front. Microbiol..

[8] Matilla M., Krell T., Egamberdieva D., Ahmad P. (2018). Plant growth promotion and biocontrol mediated by plant-associated bacteria BT. Plant Microbiome: Stress Response.

[9] Chowdhury S.P., Hartmann A., Gao X.W., Borriss R. (2015). Biocontrol mechanism by root-associated *Bacillus amyloliquefaciens* FZB42-A review. Front. Microbiol..

[10] Zehnder G., Kloepper J., Tuzun S., Yao C., Wei G., Chambliss O., Shelby R. (1997). Insect feeding on cucumber mediated by rhizobacteria-induced plant resistance. Entomol. Exp. Appl..

[11] Van der Ent S., Van Wees S.C.M., Pieterse C.M.J. (2009). Jasmonate signaling in plant interactions with resistance-inducing beneficial microbes. Phytochemistry.

[12] Song G.C., Ryu C.M. (2013). Two volatile organic compounds trigger plant self-defense against a bacterial pathogen and a sucking insect in cucumber under open field conditions. Int. J. Mol. Sci..

[13] Conrath U., Pieterse C.M.J., Mauch-mani B. (2002). Priming in plant-pathogen interactions. Trends Plant Sci..

[14] Choudhary D.K., Johri B.N. (2009). Interactions of *Bacillus* spp. and plants-With special reference to induced systemic resistance (ISR). Microbiol. Res..

[15] Van Peer R., Niemann G.J., Schippers B. (1991). Induced resistance and phytoalexin accumulation in biological control of *Fusarium* Wilt of Carnation by *Pseudomonas* sp. Strain WCS417r. Phytopathology.

[16] Pastor V., Luna E., Mauch-Mani B., Ton J., Flors V. (2012). Primed plants do not forget. Environ. Exp. Bot..

[17] Fahimi A., Ashouri A., Ahmadzadeh M., Hoseini Naveh V., Asgharzadeh A., Maleki F., Felton G.W. (2014). Effect of PGPR on population growth parameters of cotton aphid. Arch. Phytopathol. Plant Prot..

[18] Disi J.O., Zebelo S., Kloepper J.W., Fadamiro H. (2018). Seed inoculation with beneficial rhizobacteria affects European corn borer (Lepidoptera: Pyralidae) oviposition on maize plants. Entomol. Sci..

[19] Hogenhout S.A., Ammar E.-D., Whitfield A.E., Redinbaugh M.G. (2008). Insect vector interactions with persistently transmitted viruses. Annu. Rev. Phytopathol..

[20] Peccoud J., Figueroa C.C., Silva A.X., Ramirez C.C., Mieuzet L., Bonhomme J., Stoeckel S., Plantagenest M., Simon J.-C. (2008). Host range expansion of an introduced insect pest through multiple colonizations of specialized clones. Mol. Ecol..

[21] Guyomar C., Legeai F., Jousselin E., Mougel C., Lemaitre C., Simon J.C. (2018). Multi-scale characterization of symbiont diversity in the pea aphid complex through metagenomic approaches. Microbiome.

[22] Guo J., Hatt S., He K., Chen J., Francis F., Wang Z. (2017). Nine facultative endosymbionts in aphids. A review. J. Asia Pac. Entomol..

[23] Su Q., Oliver K.M., Xie W., Wu Q., Wang S., Zhang Y. (2015). The whitefly-associated facultative symbiont *Hamiltonella defensa* suppresses induced plant defences in tomato. Funct. Ecol..

[24] Oliver K.M., Degnan P.H., Hunter M.S., Moran N.A. (2009). Bacteriophages encode factors required for protection in a symbiotic mutualism. Science (80-).

[25] Frago E., Mala M., Weldegergis B.T., Yang C., McLean A., Godfray H.C.J., Gols R., Dicke M. (2017). Symbionts protect aphids from parasitic wasps by attenuating herbivore-induced plant volatiles. Nat. Commun..

[26] Angelella G., Nalam V., Nachappa P., White J., Kaplan I. (2018). Endosymbionts differentially alter exploratory probing behavior of a nonpersistent plant virus vector. Microb. Ecol..

[27] Hackett S.C., Karley A.J., Bennett A.E. (2013). Unpredicted impacts of insect endosymbionts on interactions between soil organisms, plants and aphids. Proc. R. Soc. B Biol. Sci..

[28] Dunlap C.A., Kim S.J., Kwon S.W., Rooney A.P. (2016). *Bacillus velezensis* is not a later heterotypic synonym of *Bacillus amyloliquefaciens*; *Bacillus methylotrophicus*, *Bacillus amyloliquefaciens* subsp. Plantarum and ‘*Bacillus oryzicola*’ are later heterotypic synonyms of *Bacillus velezensis* based on phylogenom. Int. J. Syst. Evol. Microbiol..

[29] Schuelke M. (2000). An economic method for the fluorescent labeling of PCR fragments. Nat. Biotechnol..

[30] Borodovsky M., McIninch J. (1993). GenMark: Parallel gene recognition for both DNA strands. Comput. Chem..

[31] Peccoud J., Bonhomme J., Mahéo F., de la Huerta M., Cosson O., Simon J.C. (2014). Inheritance patterns of secondary symbionts during sexual reproduction of pea aphid biotypes. Insect Sci..

[32] Ramos P., Rivas N., Pollmann S., Casati P., Molina-Montenegro M.A. (2018). Hormonal and physiological changes driven by fungal endophytes increase Antarctic plant performance under UV-B radiation. Fungal Ecol..

[33] Carrasco Loba V., Pérez Alonso M.-M., Pollmann S., Dandekar T., Naseem M. (2017). Monitoring of crosstalk between jasmonate and auxin in the framework of plant stress responses of roots. Auxins and Cytokinins in Plant Biology: Methods and Protocols.

[34] Manschadi A.M., Sauerborn J., Stützel H., Göbel W., Saxena M.C. (1998). Simulation of faba bean (*Vicia faba* L.) root system development under Mediterranean conditions. Eur. J. Agron..

[35] Adasme-Carreño F., Muñoz-Gutiérrez C., Salinas-Cornejo J., Ramírez C.C. (2015). A2EPG: A new software for the analysis of electrical penetration graphs to study plant probing behaviour of hemipteran insects. Comput. Electron. Agric..

[36] Sarria E., Cid M., Garzo E., Fereres A. (2009). Excel Workbook for automatic parameter calculation of EPG data. Comput. Electron. Agric..

[37] Buensanteai N., Yuen G.Y., Prathuangwong S. (2009). Priming, signaling, and protein production associated with induced resistance by *Bacillus amyloliquefaciens* KPS46. World J. Microbiol. Biotechnol..

[38] Beris D., Theologidis I., Skandalis N., Vassilakos N. (2018). *Bacillus amyloliquefaciens* strain MBI600 induces salicylic acid dependent resistance in tomato plants against Tomato spotted wilt virus and Potato virus y. Sci. Rep..

[39] Asari S., Ongena M., Debois D., De Pauw E., Chen K., Bejai S., Meijer J. (2017). Insights into the molecular basis of biocontrol of *Brassica* pathogens by *Bacillus amyloliquefaciens* UCMB5113 lipopeptides. Ann. Bot..

[40] Brock A.K., Berger B., Schreiner M., Ruppel S., Mewis I. (2018). Plant growth-promoting bacteria *Kosakonia radicincitans* mediate anti-herbivore defense in *Arabidopsis thaliana*. Planta.

[41] Xie S., Jiang H., Ding T., Xu Q., Chai W., Cheng B. (2018). *Bacillus amyloliquefaciens* FZB42 represses plant miR846 to induce systemic resistance via a jasmonic acid-dependent signalling pathway. Mol. Plant Pathol..

[42] Kloth K.J., Wiegers G.L., Busscher-Lange J., Van Haarst J.C., Kruijer W., Bouwmeester H.J., Dicke M., Jongsma M.A. (2016). AtWRKY22 promotes susceptibility to aphids and modulates salicylic acid and jasmonic acid signalling. J. Exp. Bot..

[43] Herman M.A.B., Nault B.A., Smart C.D. (2008). Effects of plant growth-promoting rhizobacteria on bell pepper production and green peach aphid infestations in New York. Crop Prot..

[44] Boutard-Hunt C., Smart C.D., Thaler J., Nault B.A. (2009). Impact of plant growth-promoting rhizobacteria and natural enemies on *Myzus persicae* (Hemiptera: Aphididae) infestations in pepper. J. Econ. Entomol..

[45] Martinuz A., Schouten A., Menjivar R.D., Sikora R.A. (2012). Effectiveness of systemic resistance toward *Aphis gossypii* (Hom., Aphididae) as induced by combined applications of the endophytes *Fusarium oxysporum* Fo162 and *Rhizobium etli* G12. Biol. Control.

[46] Gadhave K.R., Gange A.C. (2016). Plant-associated *Bacillus* spp. alter life-history traits of the specialist insect *Brevicoryne brassicae* L.. Agric. For. Entomol..

[47] Naeem M., Aslam Z., Khaliq A., Ahmed J.N., Nawaz A., Hussain M. (2018). Plant growth promoting rhizobacteria reduce aphid population and enhance the productivity of bread wheat. Braz. J. Microbiol..

[48] Pineda A., Zheng S.J., van Loon J.J.A., Dicke M. (2012). Rhizobacteria modify plant-aphid interactions: A case of induced systemic susceptibility. Plant Biol..

[49] Blubaugh C.K., Carpenter-Boggs L., Reganold J.P., Schaeffer R.N., Snyder W.E. (2018). Bacteria and competing herbivores weaken top–down and bottom–up aphid suppression. Front. Plant Sci..

[50] Stewart S.A., Hodge S., Bennett M., Mansfield J.W., Powell G. (2016). Aphid induction of phytohormones in *Medicago truncatula* is dependent upon time post-infestation, aphid density and the genotypes of both plant and insect. Arthropod Plant Interact..

[51] Oliver K.M., Higashi C.H. (2019). Variations on a protective theme: *Hamiltonella defensa* infections in aphids variably impact parasitoid success. Curr. Opin. Insect Sci..

[52] Sochard C., Morlière S., Toussaint G., Outreman Y., Sugio A., Simon J.C. (2020). Examination of the success rate of secondary symbiont manipulation by microinjection methods in the pea aphid system. Entomol. Exp. Appl..

[53] Blakeslee J.J., Spatola Rossi T., Kriechbaumer V. (2019). Auxin biosynthesis: Spatial regulation and adaptation to stress. J. Exp. Bot..

[54] Kurepin L.V., Park J.M., Lazarovits G., Bernards M.A. (2014). Burkholderia phytofirmans-induced shoot and root growth promotion is associated with endogenous changes in plant growth hormone levels. Plant Growth Regul..

[55] Kumar A., Patel J.S., Meena V.S., Ramteke P.W. (2019). Plant growth-promoting rhizobacteria: Strategies to improve abiotic stresses under sustainable agriculture. J. Plant Nutr..

[56] Kumar A., Patel J.S., Meena V.S., Srivastava R. (2019). Recent advances of PGPR based approaches for stress tolerance in plants for sustainable agriculture. Biocatal. Agric. Biotechnol..

[57] He L., Li C., Liu R. (2017). Indirect interactions between arbuscular mycorrhizal fungi and *Spodoptera exigua* alter photosynthesis and plant endogenous hormones. Mycorrhiza.

[58] Bhattacharyya P.N., Jha D.K. (2012). Plant growth-promoting rhizobacteria (PGPR): Emergence in agriculture. World J. Microbiol. Biotechnol..

[59] Radwan S.S., Dashti N., El-Nemr I.M. (2005). Enhancing the growth of *Vicia faba* plants by microbial inoculation to improve their phytoremediation potential for oily desert areas. Int. J. Phytoremed..

[60] Elbadry M., Taha R.M., Eldougdoug K.A., Gamal−Eldin H. (2006). Induction of systemic resistance in faba bean (*Vicia faba* L.) to bean yellow mosaic potyvirus (BYMV) via seed bacterization with plant growth promoting rhizobacteria. J. Plant Dis. Prot..

[61] Nalam V., Louis J., Shah J. (2019). Plant defense against aphids, the pest extraordinaire. Plant Sci..

[62] Alvarez A.E., Tjallingii W.F., Garzo E., Vleeshouwers V., Dicke M., Vosman B. (2006). Location of resistance factors in the leaves of potato and wild tuber-bearing *Solanum* species to the aphid *Myzus persicae*. Entomol. Exp. Appl..

[63] Le Roux V., Dugravot S., Brunissen L., Vincent C., Pelletier Y., Giordanengo P. (2010). Antixenosis phloem-based resistance to aphids: Is it the rule?. Ecol. Entomol..

[64] Will T., Kornemann S.R., Furch A.C.U., Tjallingii W.F., van Bel A.J.E. (2009). Aphid watery saliva counteracts sieve-tube occlusion: A universal phenomenon?. J. Exp. Biol..

[65] Paprocka M., Gliszczyńska A., Dancewicz K., Gabryś B. (2018). Novel hydroxy- and epoxy-cis-jasmone and dihydrojasmone derivatives affect the foraging activity of the peach potato aphid *Myzus persicae* (Sulzer) (Homoptera: Aphididae). Molecules.

[66] Wang W., Dai H., Zhang Y., Chandrasekar R., Luo L., Hiromasa Y., Sheng C., Peng G., Chen S., Tomich J.M. (2015). Armet is an effector protein mediating aphid-plant interactions. FASEB J..

[67] Wang W., Luo L., Lu H., Chen S., Kang L., Cui F. (2015). Angiotensin-converting enzymes modulate aphid–plant interactions. Sci. Rep..

[68] Will T., Tjallingii W.F., Thonnessen A., van Bel A.J.E. (2007). Molecular sabotage of plant defense by aphid saliva. Proc. Natl. Acad. Sci. USA.

[69] Naessens E., Dubreuil G., Giordanengo P., Baron O.L., Minet-Kebdani N., Keller H., Coustau C. (2015). A secreted MIF cytokine enables aphid feeding and represses plant immune responses. Curr. Biol..

